# Unilateral Right-Sided Progressive Flexor Digitorum Superficialis Brevis to the Little Finger: A Case Report With Review of Literature

**DOI:** 10.7759/cureus.34577

**Published:** 2023-02-02

**Authors:** Praisy Joy, Arthi G, Krati Bhardwaj, Sipra Rout

**Affiliations:** 1 Anatomy, All India Institute of Medical Sciences, Bhubaneswar, Bhubaneswar, IND

**Keywords:** fds-v, anomalous fds belly in the palm, rare variation, brevis type of fds, flexor digitorum superficialis

## Abstract

The flexor digitorum superficialis (FDS), an intermediate flexor of the forearm, can present with variations in the musculature or tendons. Here, we report a very rare anomaly of the FDS-V tendon replaced by a muscle belly in the palm, which was a progressive variation. This variation was found in a 60-year-old female cadaver on the right hand. The anomalous belly took its origin from the center of the volar aspect of the flexor retinaculum and was inserted into the A2 pulley of the middle interphalangeal joint to the little finger. The anomalous muscle was innervated by a branch of the median nerve. Knowledge of such variations will be useful for hand surgeons for meticulous planning of surgeries of the palm. The occurrences of such variations might interfere with the biomechanics of the FDS tendons.

## Introduction

The flexor digitorum superficialis (FDS) (sublimis) is an interesting muscle taking origin from two heads: humeroulnar head and radial head. The humeroulnar head originates from the medial epicondyle of the humerus and the radial head from the anterior border of the radius. The muscle belly forms the intermediate strata of muscles of the forearm and is divided into two bundles: the superficial strata, which gives two tendons, one to the middle and one to the ring finger, and the deep strata, which gives two tendons, one to the index and one to the little finger, as they approach the wrist and the tendons pass deep to the median nerve in the carpal tunnel and is inserted into the anterior surface of the middle phalanx of the two to five digits. Variations in the musculature and tendons have been described [1]. Here, we report a rare variation of FDS-V anomaly in the palm innervated by the median nerve. This report brings out the embryological and clinical significance of such FDS anomalies.

## Case presentation

**Figure 1:**
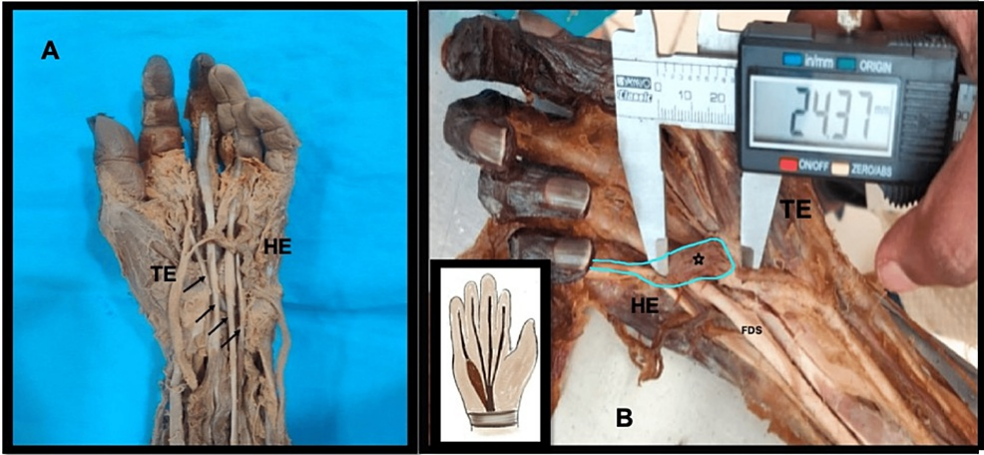
Comparison between a normal and anatomical variant of FDS brevis in the palm A. Normal left-sided palm: the arrows represent the tendons of the FDS in the palm. B: Dimensions of the FDS brevis to the little finger in situ in the right palm denoted by the star. The green outline denotes the FDS brevis muscle. FDS: flexor digitorum superficialis, HE: hypothenar eminence, TE: thenar eminence

During routine cadaveric dissection of the palm for undergraduate medical students at All India Institute of Medical Sciences (AIIMS), Bhubaneswar, we found a brevis-type insertion of the flexor digitorum superficialis to the little finger. This unique muscle variant was a part of hypothenar musculature. The tendon for the FDS was replaced by a muscle belly itself in the right palm of a 60-year-old female cadaver. The abnormal muscle belly had its origin in the palmar surface of the flexor retinaculum and its insertion into the base of the middle phalanx of the little finger. The muscle was innervated by a branch from the first common digital branch of the median nerve from its dorsal surface. The length and breadth of the muscle belly were 24.37 mm and 6.95 mm, respectively (Figure 1). It was placed obliquely between the superficial palmar arch superficially and the other flexor tendons deeply.

## Discussion

The palm is the “pandora box” for hand surgeons with such intricate structures. A diversity of anomalies has been reported by many authors in the muscles of the palm. Tan et al. classified FDS anomalies into muscle variants and tendon variants [2]. So far, 34 cases of FDS anomalies have been reported worldwide. There was a female preponderance, and most of the anomalies were observed to be reported on the right hand. About one-third of the reported cases were bilateral [3]. Bhat et al. in 2013 reviewed 32 cases of FDS anomalies and reported that 80% of the anomalies were observed in the index finger, 15% in the little finger, and 5% in the middle and ring fingers [3]. FDS anomalies in the palm could have various presentations, from asymptomatic to carpal tunnel syndrome [4,5].

Classification

Probst and Hunter (1975) were the first to classify FDS anomalies [6]. Elliot et al. classified FDS anomalies into five types [7]. Combining the first two classifications, Bhat et al. reclassified FDS anomalies in 2014 [3]. Yesilada et al. have provided the clinical classification of FDS anomalies [8]. Table 1 shows all the classifications of FDS anomalies.

**Table 1:** Different classifications of FDS anomalies FDS: flexor digitorum superficialis, FDSB: flexor digitorum superficialis brevis

Authors	Classification and types
Probst and Hunter, 1975 [6]	The common digastric form, extension of the FDS muscle into the palm, intrinsic brevis type of variation
Elliot et al., 1999 [7]	Type 1: FDS tendon attachment, type 2: muscle belly from the flexor retinaculum to the distal attachment, type 3: digastric superficial flexor muscle, type 4: distal extension of the muscle belly into the carpal tunnel, type 5: anomalies of the FDS in the forearm, like Gantzer’s muscle
Bhat et al., 2013 [3]	Type 1a: FDSB with complete replacement of the FDS tendon with muscle, type 1b: FDSB lying alongside the tendon of the FDS, type 2a: digastric type with muscle interrupting the tendon, type 2b: digastric type with muscle lying by the side of the tendon, type 3: distal extension of the FDS muscle into the carpal tunnel
Yesilada et al., 2013 [8]	Type 1: brevis type, type 2: muscle belly extending into the carpal tunnel, type 3: digastric type

Our case corresponds to type 2 according to Elliot et al. [7] and type 1b according to Bhat et al. [3].

Embryology and comparative anatomy

Phylogeny and ontogeny studies on the hand musculature reveal that there are two theories for the origin of FDS: single origin theory and dual origin theory. The dual origin theory has been widely accepted and approved by clinical anatomists. The dual origin theory claims that there are two anlages for the development of flexor digitorum superficialis: antebrachial anlage and palmar anlage. The palmar anlage migrates to the forearm and fuses with the antebrachial anlage to form the FDS muscle proper. Failure of migration of the palmar anlage results in an accessory muscle belly in the palm [9]. Table 2 shows the evolutionary variability in the development of muscles of the forearm and hand.

**Table 2:** Evolutionary variability in the development of the muscles of the forearm and hand FDS: flexor digitorum superficialis

	Tetrapods (amphibians)	Reptiles/monotremes	Mammals	Impression
Forearm	Three groups of flexor muscles: radial and ulnar flexors, and flexor digitorum communis or longus	Single flexor digitorum longus muscle in the forearm	Two layer of flexor digitorum longus and flexor digitorum profundus to the digits	The evolution of the FDS is complex in the forearm. From three layers in tetrapods, it becomes single-layered in reptiles and two-layered in mammals.
Hand	A set of superficial, short finger flexors, the flexors brevis superficialis	A set of superficial, short finger flexors, the flexors brevis superficialis	FDP tendon passing into the hand to insert on the terminal phalanges	The FDS tendon in the hand is derived from ancestral short flexors and palmaris longus.

Studies on the development of the FDS muscle in amphibians suggest that the FDS muscle is atavistic in origin. The FDS muscle has an interesting evolutionary history. Tetrapods possess three layers of muscle in the forearm, and therian mammals have only two layers of FDS. Many authors have concluded that the FDS brevis muscle is homologous to ancestral flexor digitorum superficialis. Considering the evolutionary concept, FDS brevis is thought to be an atavistic reemergence of ancestral FDS brevis muscle. However, the origin of FDS remains unclear, whether it is a muscle of ancestral FDS or the distal tendons of the FDS have been derived from another source with the FDS belly getting regressed [10]. Embryologically, FDS variation can be classified as progressive and retrogressive as mentioned by Ray et al. in 2015 [11]. Our case is of progressive type. This is a unique presentation indicating the tendency of the muscle to become functionally more complex in its evolutionary process. Progressive variation depicts the separation of the individual muscle belly of the FDS, whereas retrogressive variation depicts the occurrence of remnant connections between the two bundles of the FDS muscle [11].

Clinical manifestation of FDS variants

FDS-V variation can present with variable symptoms. The symptoms can be asymptomatic to compression neuropathies, specifically carpal tunnel syndrome. It may present as a palpable, painless mass in the palm or trigger finger. Treatment of this rare variation is based on the symptom. The treatment may be simple exploration to simple neurectomy and partial excision to complete excision depending on the symptoms. A simple neurectomy has been suggested as the procedure of choice for the digastric variation [3].

FDS brevis variation has presented as an incidental finding during cadaveric dissection to symptomatic carpal tunnel syndrome. Way back in 1927, Mainland was the first to describe such a variation in the right hand in a 45-year-old male, which had to be excised [12]. Fromont (1895) demonstrated a bilateral FDS-I anomaly by dissection on a cadaver [13]. Gräper (1917) reported a right-sided FDS-II variation by cadaveric dissection [14]. Since 2006, there has been a shift in the variations that have been reported. The FDS-V variation has been frequently reported. All five cases reported from 2006 till date have been cadaveric reports mostly having a right preponderance [3]. Our case also being unilateral on the right side is an FDS-V variation.

Kobayashi et al. reported a similar case in the right hand of an 80-year-old cadaver innervated by a branch of the median nerve. The case of Kobayashi et al. also had FDB anomaly in the foot and sternalis muscle in the trunk [9]. Our case had no such additional findings.

A genetic link has not been yet established, although there have been descriptions of FDS variations being inherited in two generations [6]. Clinical testing and imaging modalities have been used to characterize the variation. MRI would bring out the variation in 3D mode, leading to a better understanding of the pathology of compressive neuropathies in the palm [2,4,5]. Modified Baker’s test has been suggested to find out the variants of the FDS function [2].

## Conclusions

Knowledge of a specifically progressive variety of FDS will be helpful to hand surgeons to understand the etiology and plan their surgeries on hand strategically and effectively. The knowledge of such variation would be helpful for avoiding the confusion of structures during surgical approach to the palm.
